# Dementia risk among individuals with a migrant background—a scoping review

**DOI:** 10.3389/frdem.2025.1667478

**Published:** 2025-11-18

**Authors:** Felix Wittmann, Melanie Luppa, Jochen René Thyrian, Wolfgang Hoffmann, Steffi G. Riedel-Heller

**Affiliations:** 1 Institute of Social Medicine, Occupational Health and Public Health (ISAP), Leipzig University, Leipzig, Germany; 2 Institute for Community Medicine, University Medicine Greifswald (UMG), Greifswald, Germany; 3 German Centre for Neurodegenerative Disease (DZNE), Greifswald, Germany

**Keywords:** dementia, risk, prevention, migrant background, migration, refugees, review

## Abstract

**Background:**

While non-pharmacological dementia prevention is increasingly prioritized in research and policy, intersectional perspectives remain underrepresented. These are essential to address structural determinants of health and persistent diversities and inequities. This scoping review aimed to synthesize the existing amount of research on dementia risk and prevention in relation to migration background, focusing on three questions: (1) dementia risk and associated risk factors, (2) prevention, and (3) evidence concerning the most marginalized migrant populations.

**Methods:**

A systematic search was conducted in PubMed, PsycInfo, and Web of Science following PRISMA guidelines. The Population-Concept-Context (PCC) framework was used to define inclusion and exclusion criteria. Two researchers independently screened abstracts and full texts; discrepancies were resolved through discussion with a third reviewer. The included studies were synthesized and discussed.

**Results:**

Thirty-nine studies met the inclusion criteria for (1), including some known risk factors and relevant migration background factors, such as language barriers, discrimination, and mental health. Regarding prevention (2), only a few studies addressed migration-related aspects, including faith, internet use, or diagnosis. Evidence on asylum seekers, undocumented individuals, or those with irregular status was absent (3).

**Conclusion:**

This review highlights significant knowledge gaps in dementia research concerning people with a migrant background. However, risk and preventive factors were summarized and partially combined regarding targeted, sensible prevention. Nevertheless, migration might modify dementia risk across multiple levels, yet preventive efforts remain sparse. Addressing these gaps is essential to designing equitable strategies for reducing dementia risk and inclusive implementation in research and practice.

**Systematic review registration:**

identifier: OSF.IO/YHVAW.

## 1 Introduction

The trend of increasing dementia prevalence is concerning (Livingston et al., 2024). Research is steadily focusing more on lifestyle-based primary prevention at the individual level, which includes, among others, mental or socioecological aspects. According to the World Health Organization (WHO), primary prevention is defined by actions aiming to avoid the manifestation of diseases, including the improvement of health through change of social and economic determinants on health, the provision of information on behavioral and medical health risks, and other measures on the personal and community level (World Health Organization, 2025). Primary intervention, however, must directly address disparities in dementia risk (O'Donnell and O'Donnell, 2023). It is important to consider inequities, for instance, educational or socioecological inequities, as well as differences within a society, such as between genders (Roes et al., 2022). A part of society that has received little attention to date in relation to primary prevention of dementia is people with a migration background and migration experience.

Although research on risk factors and primary prevention of cognitive decline and dementia is steadily progressing, the role of migration in this context has received comparatively little attention, despite its evident relevance. So far, the relationship between dementia and migration background has primarily been examined in terms of the prevalence of dementia for people with a migrant background. Except for this, the role of migration background for individuals with diagnosed dementia regarding care and care access has not been surveyed so far (Thyrian, 2022; Monsees et al., 2022). In 2017, migrant people were first mentioned in the Global Action Plan on the public health response to dementia by the World Health Organization (WHO) (World Health Organization, 2017). But the national dementia plans of the European countries are referring to migration only marginally, if they do at all (Schmachtenberg et al., 2020). The International Organization for Migration reports 281 million international migrants globally in 2020, with 117 million people living in displacement globally at the end of 2022 (IOM UN Migration, 2024). This is an increase of 90 million people over the last 20 years and goes along with growing migration inequality. Due to the latest wars and human crisis, the number of people with a migration background is expected to increase, while demographic change will contribute to the increasing number of older people and to a change in the structure of the population, and so of people with a migration background (Canevelli and Vanacore, 2020). In Europe, one million people with a migrant background are reported to suffer from cognitive disorders (Cappa and Canevelli, 2022).

Despite broad definitions and rationales, migration appears to be a significant factor influencing individual trajectories of health and cognitive aging, based on available evidence. A first consideration here is the complexity of the subject. There are different migration phases, each with its respective factors influencing the health of migrants. In a pre-migration phase, pre-migratory events and traumata, epidemiological profiles, and cultural and geographic proximities may be relevant. During the movement phase, the duration, circumstances, and conditions of the journey can have an influence, as well as experiencing violence, exploitation, and other abuses, while in the arrival and integration phase, migration policies, status, access to services, language, and cultural values are among health-influencing factors (Wickramage et al., 2018). Certain risk factors can be pre-migration but also post-migration (Alemi and Stempel, 2018; Gleeson et al., 2020). Overall, migration can moderate the effect of risk factors. However, a migration background might be relevant for the risk of dementia. A meta-analysis about the prevalence and incidence of dementia in people with a migration background reported a higher prevalence compared to native persons (Selten et al., 2021). While mental health factors such as depression and chronic stress are found to be associated with dementia and its risk, those factors are at the same time found to be stronger among individuals with a migration background (Tribe, 2002; Bhugra, 2004). On the other side, there is very little evidence of lower prevalence rates of dementia in people with a migration background (Wändell et al., 2019). Discussing the results, however, the few studies agree on the difficulty of explaining the relationship, especially against the background of a possible underdiagnoses of dementia in people with a migratory background (Wändell et al., 2019; Stevnsborg et al., 2016).

There are certain difficulties in working with a broad framework on the subject of migration and migration background, which we would like to anticipate here. First, there are different ways of understanding migration status, especially against the background of international differences. While in some countries the term minorities is more commonly used, in other contexts, refugees, migrants, or people with a migration background are used synonymously (Sasse and Thielemann, 2005). Another relevant factor to keep in mind is the reason why people migrate. This is mostly related to the origin and can be economic and political reasons, such as persecution, demographics, or increasingly due to changing climatic conditions (Telsaç and Yüksek Telsaç, 2022; Scheel and Tazzioli, 2022). Moreover, difficulties in studying immigrant populations must be taken into account, such as misinterpretation due to cultural differences, language, or unjustifiable assumptions of homogeneity of people from a single large geographical region (Livingston and Sembhi, 2003).

Taking a closer look at the topic of migration background in relation to dementia risk and prevention is of great importance in many aspects. There may be some risk factors that differ due to migration (experience) at an individual level, such as higher depression and trauma or language barriers. Furthermore, it is relevant to examine the topic with regard to community- and population-based aspects. By focusing on risk factors and primary prevention, this review aims to assess the extent and nature of literature and the current state of research. A scoping review was chosen due to the limited and diverse evidence. Nevertheless, to maintain a clear structure, the main research objective was divided into three in-depth research questions, which are described below.

### 1.1 Review questions

To provide a comprehensive overview of the relation between risk of dementia, prevention, and migrant background, the main research question we followed for this scoping review was:

*What do we know about the risk of dementia in relation to a migrant background so far?*

The overall aim was to identify and present research carried out to date on this research objective. To ensure a clear concept and a comprehensible implementation of the scoping review, we further formulated three more in-depth research objectives:

- *What is currently known about dementia risk and associated risk factors among individuals with a migrant background?*
- *What is currently known about dementia prevention in relation to individuals with a migrant background?*
- *What is currently known about dementia risk and prevention among the most marginalized populations with a migrant background, including asylum seekers, undocumented individuals, and those with irregular migration status?*

## 2 Methods

### 2.1 Design

A scoping review was deemed appropriate to capture existing evidence and the possible breadth of research on dementia risk and prevention in individuals with a migrant background. This approach allows for the inclusion of diverse study types, which is particularly relevant given the anticipated heterogeneity in this field (Munn et al., 2018). We further aim to both synthesize existing knowledge and identify gaps in the literature.

Three databases were searched for articles and studies published until October 2025: PubMed, PsycInfo, and Web of Science. Previously, a preliminary search of PubMed, the Cochrane Database of Systematic Reviews, and JBI Evidence Synthesis was conducted. While one relevant protocol was identified on OSF in 2022, no associated publication could be found. Furthermore, we identified one published review focusing exclusively on the prevalence of dementia in the migration population (Selten et al., 2021), and another that examined selected dementia-related factors in the Australian context (Hamrah et al., 2023). To our knowledge, no comprehensive review to date has addressed both the risk and prevention of dementia in the context of migration. In the next step, a preliminary search for respective research questions was undertaken to identify potentially relevant keywords and terms for developing a final search strategy across all databases. An overview of the final search strings is listed in Supplementary Table S1. The strings for each research objective were used across all databases and were adjusted to the interface, respectively. Furthermore, gray literature was screened (OpenGrey, Bielefeld Academic Search Engine (BASE), Google Scholar, and Google) as well as reference lists of all included sources of evidence (World Health Organization, 2017).

**Figure 1:**
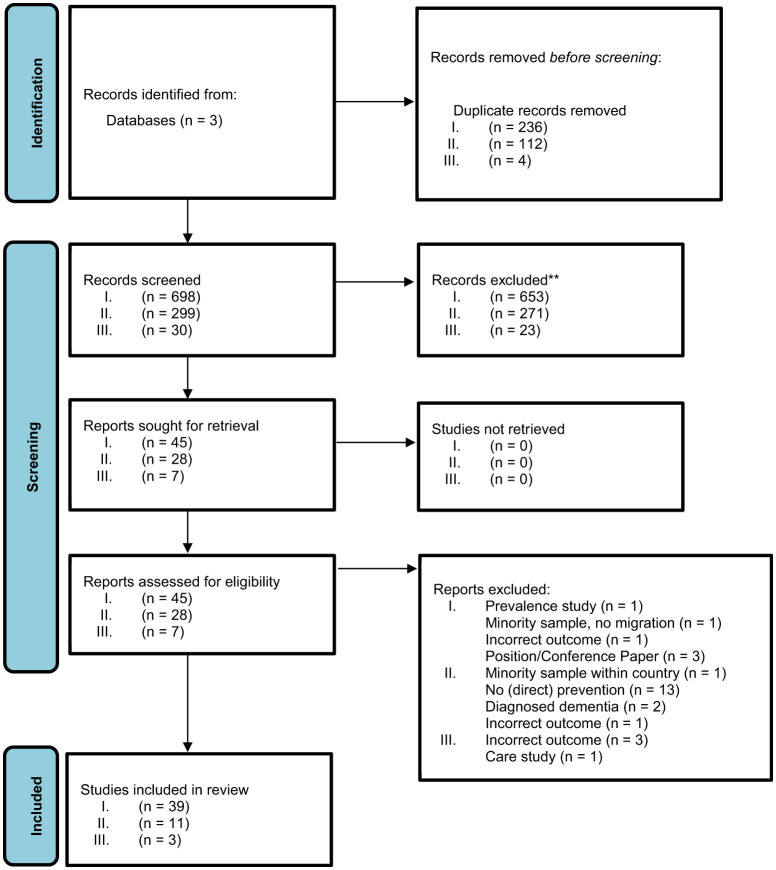
Prisma flowchart according to Tricco et al. (2018).

Following a pilot screening to identify duplicates, two independent reviewers (FW, JK) screened for assessment against the inclusion criteria for the Review. Potentially relevant sources were retrieved in full for eligibility. Any uncertainties and disagreements that arose between the reviewers at each stage of the selection process were resolved through discussion with a third reviewer (ML). The results of the systematic search are presented in a PRISMA flow diagram (see Figure 1), included studies are extracted regarding key data and findings, while the content and results of included studies are synthesized and discussed.

Since risk and prevention in relation to migration background encompass a broader spectrum of possible factors, the socio-ecological model (SEM) was used to better structure the results. The SEM is widely used, among others for prevention and health promotion and classifies factors according to respective socio-ecological levels at individual, interpersonal, community, and policy levels (Kilanowski, 2017).

In order to achieve a clear structure, methodological fineness, and completeness, the PRISMA Guidelines were used (Tricco et al., 2018). The PRISMA ScR Checklist is available in Appendix. Moreover, the scoping review has been preregistered at Open Science Framework (OSF; Wittmann, 2025).

### 2.2 Study selection

For the selection of studies, we followed the Population, Concept, and Context (PCC) framework according to Peters et al. (2022). The PCC framework enables the determination of goals and specific research questions as well as the specification of study characteristics regarding inclusion and exclusion (see Table 1).

**Table 1:** Review eligibility criteria based on the PCC framework.

	**Inclusion**	**Exclusion**
Population	• Individuals with a migration background by being born in another country [or having at least one parent who was born in a foreign country] • People related to individuals with a migrant background, e.g., Parents, General practitioners, …	• Studies without people with a migration background • Children and adolescents
Concept/types of evidence	• All sorts of empirical studies (trials, longitudinal and cross-sectional studies, qualitative studies, retrospective) • Reviews • Non-empirical evidence (discussion paper, NGO reports, short notes, letters to the editor, or comments on studies)	• Preprints • Editorials • Conference Abstracts or posters • Dissertations • Articles that are not available or not written in English or German
Context	• Dementia, cognitive impairment, or decline • Any cultural factors, geographical location, or gender stratification, and study setting I. Risk, risk factors, predisposition, or vulnerability to dementia II. Prevention of dementia III. Regarding asylum seekers, irregular or undocumented migrants, and dementia	• Other health-related outcomes • Prevalence studies without reference to other factors and indicators • Caregiver studies • Biomedical studies

Framework based on (Peters et al. 2022); I–III according to the Research Objectives mentioned in Section 2.2.

A flow diagram of screened, retrieved, and included studies was developed (see Figure 1).

## 3 Results

### 3.1 Study characteristics

After removing duplicates, 1,027 studies were screened across all three research questions. Of the screened studies, 53 studies were then included, the majority of which related to the first research question on risk and risk factors. A detailed list including stratification for research questions, including reasons for exclusion, can be found in the flowchart in Figure 1. All included studies are listed in detail in Table 2.

**Table 2:** Table to summarize details of included studies.

**Authors (year)**	**Study type**	**Country/area, city**	**Study goal**	**Study sample(s)**	**Outcome measures^a^**	**Key findings^b^**	**Risk factor**
**Research question 1**							
(Garcia et al. 2017)	Longitudinal	U.S., Texas, Galveston	Explore differences in the incidence of cognitive impairment due to nativity and age of migration	*N* = 2,708 Mexican Americans	Cognitive impairment	- Late-life immigrant women had a higher risk of cognitive impairment compared to U.S.-born women - Midlife immigrant men had a 29% lower risk of cognitive impairment	Age at migration
(Moon et al. 2019)	Cross-sectional	U.S., Kentucky, Louisville	Examine the role of immigrant status and the relation between race/ethnicity and risk of dementia	*N* = 7,609 non-Hispanic white (NHW), non-Hispanic black (NHB), Hispanic, and other Medicare beneficiaries	Dementia status (based on cognition, validated proxy-reported assessment of dementia, and a battery of cognitive tests)	- Significant interaction between immigrant status and ethnicity regarding dementia status	Immigration status
(Wong and Soong 2024)	Longitudinal/prospective cohort data	U.S., New York, Syracuse	Analyze how nativity and neighborhood interact to affect dementia risk	*N* = 5,362 U.S. adults, foreign-born vs. native-born	Time to dementia diagnosis	- Foreign-born older adults had a 51% higher risk for dementia - No significant interaction for nativity with neighborhood physical disorder or social cohesion	Neighborhood physical disorder; social cohesion
(Kezios et al. 2023)	Longitudinal	U.S., New York City	Compare conventional and propensity score-based methods to examine differences in cognitive trajectories between different subgroups of the Study sample	*N* = 22,584, including foreign-born Mexican American, U.S.-born Mexican American, and U.S.-born non-Hispanic white individuals	Global cognition	- Baseline cognitive scores were worse in Individuals of Mexican background, but cognitive decline was slower compared to non-Hispanic white Individuals - Adjusting for selection bias and AD risk factors did not account for the paradoxical finding	Migration selection factors
(Weden et al. 2017)	Longitudinal	U.S., California, Santa Monica	Evaluate cognitive trajectories of different groups to understand racial-ethnic disparities in AD, investigating whether factors related to migration selection explain the Hispanic Paradox	N = 8,433 U.S.-born non-Hispanic whites (US-NHW), U.S.-born non-Hispanic blacks (US-NHB), U.S.-born Mexican Americans (US-MA), and foreign-born Mexican Americans (FB-MA)	Cognitive measures/presence of CI	- Disadvantages in minorities due to individual and neighborhood SES are at significantly higher risk for poor cognitive aging	SES disadvantage
(Bridi et al. 2023a)	Qualitative	U.S., California, San Diego, and Amman, Jordan	Address the literature gap regarding the role of faith on mental health and cognition	N = 61 Arab. migrants	Qualitative evaluation based on Leventhal's Self-Regulation Model	- Participants report experienced impairment of cognitive functioning, including forgetfulness, reduced executive function, and inability to focus - Reasons mentioned were trauma, social isolation, violence, loss of socioeconomic status, and restrictive policies	Faith and Spirituality (protective)
(Holwerda and Hoogendijk 2020)	Non-empirical study (invited commentary)	The Netherlands, Amsterdam	Commentary	Parents who stayed in Mexico, with migrating adult children	n. a	Highlighting the importance of including family member migration status when discussing social isolation, loneliness, and health outcomes in later life	Loneliness (family member migration status)
(Downer et al. 2018b)	Longitudinal	U.S., Texas, Galveston	Examine if older adults with one or more adult children who emigrated are more likely to develop CI	*N* = 2,609, older parents in Mexico who have an adult child living in the U.S.	Cognitive impairment, SES, and depressiveness. and social engagement	- No difference in odds for developing CI when having one or more adult children emigrated but lower SES, higher depressiveness, and greater social engagement, which may affect the risk of dementia	Family member migrant status, lower SES, and depression
(Daniel et al. 2024)	post, correlational design	U.S., Florida, Boca Raton	Examine the risk of ADRD and age, sex, education, and knowledge about AD as predictors	*N* = 55 older Afro-Caribbean's with 10 years or less of education residing in a rural area within the last 20 years	Cognition: Basic knowledge of Alzheimer's disease survey (BKAD)	- Language scores and education are strongly associated with cognition - Short intervention increased basic knowledge of ADRD	Language, education
(Kaki et al. 2025)	Qualitative	U.S., California, San Francisco	Investigate migrant perspectives on dementia and access to cognitive healthcare	*N* = 69 Arab, African, and Afghan migrants in San Diego, California	Qualitative evaluation regarding the socioecological model of health	- Shared experiences, knowledge gaps, and barriers to healthy aging for a heterogeneous sample - Mental trauma, fear of dementia, limited support, stress of local community loss, and discriminatory immigration were reported as important factors	Mental traumata, limited support, Stress, Discrimination
(Zahodne et al. 2014)	Longitudinal	U.S., New York City	Examine a possible protective effect of bilingualism on age-related cognitive decline/dementia over 23 years	*N* = 1,067 Hispanic immigrants living in a Spanish-speaking enclave of northern Manhattan	Cognitive decline (episodic memory, language, executive function, speed)	- Bilingualism did not protect from cognitive decline or dementia	Bilingualism (protective factor, unconfirmed)
(Nguyen et al. 2022)	Cross-sectional	U.S., California, Davis	Examine job exposure (neurotoxins, etc., among nail salon technicians) among Vietnamese immigrant women	*N* = 155 female Vietnamese migrants, nail technicians, 145 control group participants	Cognition (MoCA) and Depression (CES-D)	- Cognitive functioning was higher for the control group compared to the nail technician group - Exposure is negatively associated with cognitive functioning	Occupational exposure to neurotoxins, Depressive symptoms
(Jang et al. 2021)	Cross-sectional	U.S., California, Los Angeles	Examine the association among social isolation, loneliness, and cognitive impairment, focusing on loneliness as a mediator	*N* = 2,061 Korean immigrants	Objective (MMSE) and subjective (single-item self-rating scale) of cognitive impairment	- Objective cognitive impairment associated with social isolation, not with loneliness - Subjective cognitive impairment associated with social isolation and loneliness - Potential mediating role of loneliness between social isolation and subjective cognitive impairment	Social isolation, Loneliness
(Vásquez et al. 2022)	Longitudinal	U.S., New York, Albany	Examine diabetes and age at migration as risk factors	*N* = 3,050 Mexican-origin older adults	Cognition (MMSE)	- Diabetes is associated with a higher risk of developing dementia - When immigrants migrated at age 50 years or older, a higher dementia risk was found	Age at migration, Diabetes
(Casanova and Aguila 2020)	Cross-sectional	U.S., California, Fullerton	Examine gender differences among Mexican Immigrants	Mexican-born older adults residing in the United States	Cognitive functioning	- After controlling for socioeconomic factors, no difference between male Mexican immigrants and non-immigrants, but for women - Hypothesis, that this is due to the underrepresentation of female Mexican immigrants in terms of predisposition to cognitive decline	Gender (assumption of selection hypothesis)
(Kovaleva et al. 2021)	Literature update	U.S., Tennessee, Nashville	Summarize factors associated with immigrants' risk and care, interventions, and nurse strategies	Older immigrants in the U.S.	Literature update	- Socioeconomic, health literacy, cultural, psychological wellbeing, and English language proficiency as factors associated with cognitive impairment among immigrants - Call for changes in healthcare to meet needs, more targeted and professional support	Language barriers, Economic constraints, Depressiveness, Social isolation, Low acculturation, Stigma
(Tang et al. 2023)	Longitudinal	U.S., Pennsylvania, Pittsburgh	Examine age at migration, reasons for migration, acculturation, perceived discrimination, and association with cognition	*N* = 2,075 Chinese immigrants	Cognition	- Perceiving discrimination and lower levels of acculturation are associated with faster decline	Perceiving discrimination, Lower level of acculturation
(Guo et al. 2022)	Cross-sectional	U.S., Iowa, Iowa City	Examine the role of living in an ethnic enclave and cognition	*N* = 3,105 Chinese Elderly in Chicago; of these, N = 1,870 living in an ethnic enclave (Chinatown)	Cognition (MMSE; working memory, episodic memory, executive function)	- Living in an ethnic enclave was found to be a risk factor for poor cognition - Education plays a salient role in explaining the cognitive disadvantage of ethnic enclave residents	Living in an ethnic enclave
(Haan et al. 2011)	Longitudinal	U.S., California, San Francisco	Examine the effect of lifetime SES trajectories and cumulative disadvantage from childhood on late-life cognition	*N* = 1,789 Mexican Americans	Cognitive performance (3MSE, SEVLT)	- More advantaged lifetime SES trajectories are associated with fewer declines compared with low SES over the life course - Intergenerational changes in SES, cultural norms, behaviors, and changes in environment might explain the heterogeneity of cognitive aging among diverse ethnic groups	Lifetime SES trajectories, Cumulative disadvantage across lifespan
(Kindratt et al. 2024)	Cross-sectional	U.S., Texas, Arlington	Examine differences in risk factors (education, hearing loss, traumatic brain injury, hypertension, alcohol use, obesity, smoking, depression, marital status, physical inactivity, and diabetes) between different groups	*N* = 108,695 Middle Eastern and North African American immigrants compared to U.S.- and foreign-born non-Hispanic White adults	risk factors	- Higher odds for less than 9th grade education, psychological health concerns, and diabetes among immigrants	Education, Hearing loss, Traumatic brain injury, Hypertension, Alcohol use, Obesity, Smoking, Depressiveness, Marital status, Physical inactivity, Diabetes
(Hamrah et al. 2023)	Systematic review	Australia, Tasmania	Identifying studies analyzing modifiable risk factors within Australia	Individuals with a migrant background in Australia	Studies about risk factors only, no further association with dementia/cognitive functioning	- Higher prevalence of depression, physical inactivity, diabetes, and social isolation - Risk factors associated with demographic characteristics, culture of origin, life course events, country of birth, age at arrival, and length of stay	Depression, Social isolation, Physical inactivity, Diabetes
(Prakash 2011)	Comment	India, New Delhi	Comment	Older UK African–Caribbean population	n. a	Summarizing articles regarding the older UK African-Caribbean population, querying the etiology of vascular factors as primary to the increased prevalence of dementia	Depression, Illiteracy
(Kim et al. 2023)	Longitudinal	U.S., California, San Francisco	Examine the effect of place-based factors on dementia in individuals with a migrant background	*N* = 9,854 migrants aged 40+ years upon arrival in Denmark	Dementia (composite outcome) and all-cause mortality	- No association was found between neighborhood disadvantage and dementia, either for the migrant or for the non-migrant sample	Neighborhood disadvantage
(Singh et al. 2023)	Longitudinal	Canada, London	Identify characteristics of an individual that are of greatest relevance for healthy cognitive function in mid to late life	Representative Canadian Sample (Canadian Longitudinal Study Aging)	Cognitive function (normalized latent construct score)	- Among different categories, demographics and socioeconomics were most associated with cognitive functioning - Immigration, being white vs. non-white, urban vs. rural, and income were the strongest predictors associated with cognition	Immigration status, Nutritional risk, Community belonging
(Tang et al. 2019)	Longitudinal	U.S., Pennsylvania, Pittsburgh	Examine the association between immigration-related factors and cognitive impairment	*N* = 2,443 adults aged 60 and older who self-identified as Chinese	Cognition (C-MMSE)	- Women were more likely to have CI than men - None of the immigration-related factors were related to CI	Immigration experience (acculturation, discrimination)
(Simon et al. 2023)	Perspective review	U.S., New York City	Identifying research gaps regarding health inequities and promotion of mental and brain health in Brazilian immigrants	Brazilian immigrants in the U.S.	n. a	- List of recommendations to promote greater inclusion of the US Brazilian populations - i.e., epidemiological studies to map prevalence and incidence of mental/brain health conditions	N. a
(Franco and Choi 2020)	Cross-sectional	U.S., California, Los Angeles	Investigating whether immigrant status is linked to greater odds of dementia/undiagnosed dementia	*N* = 7,385 adults aged 65 years and older (6,567 U.S.-born and 818 foreign-born)		- Older immigrants had greater odds of having dementia - Immigrant status was associated with a greater risk of undiagnosed diseases like diabetes, hypertension, and hyperlipidemia - Language proficiency of relevance for the prevalence of dementia	Immigration status, Language proficiency
(Downer et al. 2018a)	Longitudinal	U.S., Texas, Galveston	Examine the role of education for the risk of cognitive impairment by nativity, age of migration, and gender	*N* = 3,424 Mexican American adults aged 65 and older living in Texas, Arizona, California, Colorado, and New Mexico	Cognitive functioning (MMSE)	- Foreign-born women and men had a higher risk for cognitive impairment, whereby only for women the association remained when adjusting for education - Education was found to be a mediator for the association between age of migration and CI	Age of migration, Education as mediator
(Luo et al. 2023)	Cross-sectional	U.S., North Carolina, Greenville	Assess the association between poor oral health and mild cognitive impairment, and the potential modifying effect on this association by age of immigration	N=5,709 Hispanic/Latino immigrants	MCI (test battery)	- Immigrants with significant tooth loss were more likely to have MCI - Migration to the U.S. after the age of 18 was associated with greater odds of MCI than migration at a younger age - An interaction effect between tooth loss and age of immigration was found to affect MCI between 35 and 49 years	Age of migration, oral health
(Meyer et al. 2024)	Longitudinal/study protocol	U.S., California, Davis	Study protocol for a longitudinal study examining the history of war and trauma and cognition over 2 years	*N =* 548 Vietnamese-Americans	Cognitive assessments and blood samples	- Study protocol, no results yet - Authors expect to provide insights into an understudied group, including traumata and the history of war	Early life adversity, War-related trauma
(Saadi et al. 2021)	Cross-sectional	U.S., Massachusetts, Boston	Examine the association between trauma exposure/PTSD and CI among immigrants; assess whether sleep quality attenuates the PTSD-CI association	Individuals of Hispanic/Latino background and Asian/Pacific Islander adults aged 60 or older	MCI (MoCA)	- Higher PTSD was associated with decreased odds of normal cognitive functioning in the Asian, not the Latino group - Daytime sleepiness did not moderate this association	Traumata, PTSD, Sleep quality
(Fuller-Thomson et al. 2020)	Cross-section (longitudinal design, but using just baseline data)	Canada, Toronto	Examine the healthy immigrant effect for verbal fluency	*N* = 8,574 Immigrants (recent and long-term) and Canadian-born residents	Verbal fluency (Controlled Oral Word Association Test and Animal Fluency)	- Long-term immigrants had higher VF than Canadian-born counterparts - Immigrant status, social, health, and nutritional factors are important for possible intervention and prevention strategies for CI	Immigration status, social factors, nutrition
(Torres et al. 2020)	Longitudinal	U.S., Texas, Galveston	Evaluate the association between adult child migration status and change in cognitive performance	*N* = 5,972 Mexican individuals with at least one child emigrated	Cognitive performance (verbal memory scores, overall cognitive performance)	- An association between an adult child who emigrated and a decline in verbal memory scores and overall cognitive performance was found for women, not for men	Family member migration status
(Hamrah et al. 2025b)	Cross-sectional	Australia, Tasmania	Compare modifiable risk factors among South Asian migrants and non-migrants	*N* = 136 South Asian migrants and *N* = 2,743 non-migrant Australians aged 50 or older	Risk factors	- Different risk profiles between the groups - Need for targeted interventions for specific risk groups	Hypertension, Hypercholesterolemia, Cognitive inactivity, Smoking, Mediterranean-type diet, alcohol consumption
(Oaxaca Carrasco et al. 2025)	Cross-sectional	Mexico	Examine the effect of migration experience, (un)documented status, and informal sector participation	*N* = 6,277	Cognitive status (Cross-Cultural Cognitive Examination, Informant Questionnaire on Cognitive Decline in the Elderly)	Undocumented return migrants working in the informal sector have worse cognitive outcomes than non-migrants and undocumented return migrants in the formal sector	Migration experience, participation in the formal or informal sector
(Kim et al. 2024)	Cross-sectional	U.S., Texas	Examine the association between social isolation and cognitive health, including dietary risk as a mediator	*N* = 2,061 older Korean American immigrants aged 60 and older	MMSE	- Social isolation was associated with cognitive health - Dietary habits and practices were found to be mediators	Social isolation, Diet
(Chen et al. 2025)	Longitudinal cohort study	U.S., Chicago metropolitan area	Identify multiple internal and external characteristics of stress and their interrelatedness and influence on age-associated cognitive decline	*N* = 1,528 older Chinese Americans aged 60 and older	MMSE, East Boston Memory Test, Digit Span Backward, Oral Symbol Digit Modalities Test	- Factor analysis identified three main constructs of behavioral and sociocultural aspects: stress internalization, neighborhood/community cohesion, and external stress alleviation - Stress processing, hopelessness, and lower conscientiousness were associated with a decline in memory	Stress internalization, Neighborhood/community cohesion, External stress alleviation, and predictors of age-associated cognitive decline
(Lindhout et al. 2025)	Cross-sectional	The Netherlands, Amsterdam	Comparison of midlife dementia risk scores between ethnicities	*N* = 2,978 Dutch, *n* = 2,084 South-Asian Surinamese, *n* = 3,135 African Surinamese, *n* = 1,699 Ghanaian, *n* = 2,000 Turkish participants	Risk scores	Migrant populations had higher dementia risk scores, independent of age, than Dutch natives	Cardiovascular Risk Factors, Aging and Incidence of Dementia (CAIDE), LIfestyle for BRAin Health (LIBRA), Australian National University-Alzheimer's Disease Risk Index (ANU-ADRI)
(Hamrah et al. 2025a)	Cross-sectional	Australia, Tasmania	Describe modifiable dementia risk factors in South Asian migrants by Country of Birth	*N* = 146 South Asian participants, *n* = 54 from Bhutan, *n* = 52 from Nepal, *n* = 22 from Afghanistan, *n* = 18 from India	Risk factors	Risk factors differed by country of origin, highlighting the importance of data stratification within migrant groups to ensure more precise and targeted interventions	Hypertension, Hypercholesterolemia, Diabetes, Alcohol consumption, BMI, Cognitive Activity, Diet, Physical Activity, Smoking
**Research question 2**							
**Authors (year)**	**Study type**	**Country/area, city**	**Study goal**	**Study sample(s)**	**Outcome measures** ^a^	**Key findings** ^b^	
(Bridi et al. 2023b)	Qualitative	U.S./California, San Diego	Examine attitudes toward dementia and cognitive healthcare among migrants in Jordan	*N* = 32 older (≥ 55 years) Syrian migrants in Jordan	Semi-structured interviews in focus groups	- Participants are convinced that a healthy lifestyle can prevent dementia - Attitudes were structured on the socioecological levels of the individual, interpersonal, community, and institution/policy level	
(Tillmann et al. 2019)	Cross-sectional	Germany/Bonn	Examine the state of German GPs regarding diagnosis for Individuals with a migrant background	*N* = 982 GPs	Self-developed, written, standardized questionnaire	- GPs report having had barriers at least once (96%), uncertainties in diagnosing dementia (71%), the most frequent were language barriers (89%) and information deficits (59%)	
(Neter and Chachashvili-Bolotin 2022)	Cross-sectional	Israel/Emeq Hefer	Examine ethnic differences in attitudes and preventive behaviors to AD in Israel	*N* = 1,198 older adults: long-term Israeli Jews (LTIJ), immigrants from the Former Soviet Union (FSU), and Palestinian Citizens of Israel (PCI)	Attitudes and preventive behavior among different cultural and ethnic minorities	- Attitudes toward AD were most negative among FSU, while preventive behavior was highest among FSU - Knowledge toward health behavior is malleable, but must take into account a culturally sensitive and psycho-educational view	
(Bridi et al. 2023a)	Qualitative	U.S./California, San Diego	Examine the role of faith on mental health and cognitive health	*N* = 61 Arab migrants in San Diego, California, United States (*N* =29) and Amman, Jordan (*N* = 32).	Semi-structured interviews and focus groups were analyzed and organized based on Leventhal's Self-Regulation Model	- Faith and spirituality are of relevance for illness representation and coping procedures of mental and cognitive health in Arab refugees - Study used the Leventhal Self-Regulation Model for evaluation based on illness identity, timeline, consequences, causes, and controllability	
(Teixeira-Santos et al. 2023)	Longitudinal	Luxembourg/Esch-sur-Alzette	Examine the effectiveness of a mindfulness-based stress reduction (MBSR) vs. a Health promotion program	*N* = 90 Portuguese-speaking migrants	Executive functioning, physiological stress measures, self-reported questionnaires, qualitative interviews	Study protocol, no study was found about the final results	
(Sadarangani and Murali 2018)	Integrative review	U.S./New York City	Identify barriers and facilitators of Adult Day Service Centers (ADS) and examine the impact on older adult immigrants' health and wellbeing	Older adult Individuals with a migrant background	ADS use, functional impairments, gender, degree of loneliness, ethnicity	- ADS use differed for functional impairment, ethnicity, gender, and degree of loneliness - ADS enhanced the quality of life of immigrants, provided fulfillment - Transportation, bilingual nurses, peer support, and cultural activities were essential	
(Daniel et al. 2024)	Post, correlational design	U.S./Florida, Boca Raton	Investigate dementia risk, knowledge about AD, and potential predictors	*N* = 55 older Afro-Caribbeans with 10 years or less of education residing in a rural area within the last 20 years	Basic Knowledge Alzheimer's Disease Survey (BKAD), Mini-MoCA, MoCA-brief	- Association between Mini-MoCA and Language score and education - Significant change from pre- to posttest in BKAD after brief educational intervention regarding health-promoting factors	
(Tran et al. 2025)	Qualitative	U.S., New York City	Examine the beliefs about the causes of Alzheimer's Disease	*N* = 216 Latino immigrants aged 40 to 64	Basic knowledge and beliefs about Alzheimer's Disease	- Genetics was mentioned as the most endorsed cause of AD, but no one attributed AD to the normal process of aging - Uncertainty about the exact causes of AD shows big potential for educational interventions and improving community knowledge - Relevance of cultural competency in healthcare	
(van Ta Park et al. 2025)	Intervention study, pre- and post-test evaluation	U.S.	Evaluate the effectiveness of a dissemination workshop regarding knowledge, attitudes, and behavior regarding ADRD	*N* = 345 Korean American participants	Knowledge, Attitudes, and Preventive Behavior	- Community engagement dissemination project with two groups: workshop (in person) and online participation - Significant changes in posttest ADRD knowledge, attitudes, and behaviors among workshop participants - positive impact on improving knowledge, attitudes, and behavior regarding ADRD among Korean Americans with a culturally tailored program	
(van der Endt et al. 2025)	Intervention study	The Netherlands, Amsterdam	Examine the effectiveness of self-managing lifestyle modification with remote coaching	N=692 (aimed)	Composite score of systolic blood pressure, non-high-density lipoprotein cholesterol, and BMI	Study protocol, no study was found about the final results	
(Iziduh et al. 2025)	Theoretical review	Canada	Synthesize research on culturally-safe strategies to enhance knowledge and awareness of dementia risk among diverse women	Immigrant and ethno-culturally diverse women	Design and implementation of culturally safe dementia risk reduction strategies for immigrant women	−17 studies were included (2006–2021) - No study focused solely on women - Strategies found in the review increased knowledge of dementia and misconceptions, but did not prompt seeking dementia screening	
**Research question 3**							
(Simon et al. 2023)	Non-empirical study (perspective review)	U.S./New York City	Identifying research gaps regarding health inequities and promotion of mental and brain health in Brazilian immigrants	Brazilian immigrants in the U.S.	Studies about the prevalence and incidence of mental/brain health conditions and risk factors among the Brazilian population in the US	- Often unclear whether undocumented migrants are included in the migration quote - Stressing the need for clear differentiation and more evidence	
(Oaxaca Carrasco et al. 2025)	Cross-sectional	Mexico	Examine the effect of migration experience, (un)documented status, and informal sector participation	*N* = 6,277	Cognitive status (Cross-Cultural Cognitive Examination, Informant Questionnaire on Cognitive Decline in the Elderly)	- The study examined the migration experience of people returning to Mexico - Challenge faced by undocumented return migrants due to legal precarity, economic insecurity, and limited institutional support - especially for return migrants working in the informal sector	

AD, Alzheimer's disease; ADRD, Alzheimer's Disease and related dementia; ADS, Adult Day Service Centers; ANU-ADRI, Australian National University Alzheimer's Disease Risk Index; CAIDE, Cardiovascular Risk Factors, Aging and Incidence of Dementia; CI, Cognitive impairment; GP, General practitioner; LIBRA, LIfestyle for Brain Health; MCI, Mild cognitive impairment; MoCA, Montreal Cognitive Assessment; PTSD, Post traumatic stress disorder; SES, Socioeconomic status; U.S., United States.

^a^Outcomes extracted in this table were concentrated to outcomes regarding cognitive health only, even if several outcomes were examined in studies.

^b^If included studies are eligible for several research objectives, key findings were adapted to the respective objective.

### 3.2 Risk and risk factors

In total, 39 studies were included to evaluate research question one about what is currently known about dementia risk and associated risk factors among individuals with a migration background. Of those, 15 studies were cross-sectional (Nguyen et al., 2022; Jang et al., 2021; Casanova and Aguila, 2020; Guo et al., 2022; Kindratt et al., 2024; Franco and Choi, 2020; Luo et al., 2023; Saadi et al., 2021; Fuller-Thomson et al., 2020; Moon et al., 2019; Hamrah et al., 2025a,b; Lindhout et al., 2025; Oaxaca Carrasco et al., 2025; Kim et al., 2024), 15 studies were longitudinal (Garcia et al., 2017; Wong and Soong, 2024; Kezios et al., 2023; Weden et al., 2017; Downer et al., 2018b; Zahodne et al., 2014; Vásquez et al., 2022; Tang et al., 2023; Haan et al., 2011; Kim et al., 2023; Singh et al., 2023; Tang et al., 2019; Downer et al., 2018a; Torres et al., 2020; Chen et al., 2025) with one short Pre-Post study (Daniel et al., 2024) and one study protocol (Meyer et al., 2024). Two studies used qualitative approaches (Kaki et al., 2025; Bridi et al., 2023a). Two comments on research were included (Holwerda and Hoogendijk, 2020; Prakash, 2011), one literature update (Kovaleva et al., 2021), a systematic and a perspective review (Hamrah et al., 2023; Simon et al., 2023). While 30 studies were from America, two longitudinal studies were from Canada (Fuller-Thomson et al., 2020; Singh et al., 2023), one comment was from India (Prakash, 2011), and one comment and one study were from the Netherlands (Holwerda and Hoogendijk, 2020; Lindhout et al., 2025) and one review from Australia was included (Hamrah et al., 2023). One study came from Mexico (Oaxaca Carrasco et al., 2025) and two others from Australia (Hamrah et al., 2025a,b). Study samples were more heterogeneous, but mainly focused on Individuals with a Hispanic background, mostly Mexico-related. Some studies included Asian origins, mostly people from China and just a few included other origins or foreign-born in general. Half of the studies were published in the last 3 years and only a quarter are older than 7 years with the oldest study from 2011.

On an individual level, 17 studies reported risk factors for dementia in relation to migration background. A recent study by Lindhout et al. used three different risk scores [Cardiovascular Risk Factors, Aging and Incidence of Dementia (CAIDE), Lifestyle for BRAin Health (LIBRA), and Australian National University-Alzheimer's Disease Risk Index (ANU-ADRI)] to find that the risk scores were higher for various migrant populations than for the non-migrant population, in this case, the native Dutch population (Lindhout et al., 2025). Four studies reported age at migration as a risk factor. Respectively, among older people with a migration background, the higher the risk of developing cognitive decline. In this regard, different ages were reported: higher risk was found with migration at 50 years or older (Luo et al., 2023; Vásquez et al., 2022), also in reference to 18–34 year old immigrants and even higher compared to people who immigrated at 18 years or younger (Luo et al., 2023). One study found this effect only for women, assuming a bias due to health selectivity (Garcia et al., 2017). Education was found to mediate the association between age of migration and risk of cognitive impairment (Downer et al., 2018a). All four studies reported results of individuals with a Hispanic background. Gender as a factor of risk was taken up by three studies: One study reported higher risk for immigrated women but not for men. This was partly explained by the selection hypothesis, which indicates less positive selection of female immigrants than their male counterparts (Casanova and Aguila, 2020). A higher risk for women was also found in other studies, not linked to immigration experience (Tang et al., 2019) but mediated by education, which was not found for men (Downer et al., 2018a). Socioeconomic factors as such were reported by two studies only: it was found to be lower in migrant subgroups linked to risk of cognitive impairment (Singh et al., 2023). This makes health care access more difficult as well as health-promoting lifestyle as direct consequences (Kindratt et al., 2024).

A more frequently discussed risk factor associated with cognition was language and language proficiency (Daniel et al., 2024). Increased depression due to poorer language skills, a higher tendency to live in insular settings, or the difficulty of diagnosis due to poorer understanding are among the possible causes (Kovaleva et al., 2021). One study explicitly found that limited language proficiency mediated the association between immigration status and (undiagnosed) dementia (Franco and Choi, 2020). However, bilingualism does not appear to protect against cognitive decline, even over a period of 23 years (Zahodne et al., 2014).

Few studies were found regarding physical health. One study reported lower odds for hypertension, obesity, and hearing loss for people with a Middle Eastern or Northern African background (Kindratt et al., 2024), which was mainly supported by findings from a systematic review focused on Australia (Hamrah et al., 2023). A more frequently surveyed factor was diabetes, well-known as a risk factor for dementia, and in most studies found with a higher prevalence among people of a migration background (Vásquez et al., 2022; Hamrah et al., 2023; Kindratt et al., 2024). An association between significant tooth loss and MCI was also reported. According to the study, significant tooth loss in people with a Hispanic migration background was considered a risk factor for MCI, with a modifying effect of age at migration (Luo et al., 2023).

Mental health was addressed in several studies. The most surveyed risk factor was depression, which was found to be more prevalent in people with a migration background in general (Kindratt et al., 2024; Hamrah et al., 2023). Therefore, possible explanations are immigration as a major stressor, changes in culture, and few social interactions (Kovaleva et al., 2021). A study examining behavioral and sociocultural constructs found that stress internalization is a significant risk factor for age-associated cognitive decline, including greater perceived stress, greater hopelessness, and lower conscientiousness (Chen et al., 2025). Besides depression, anxiety was also found to be more prevalent in individuals with a Middle Eastern or North African migration background compared to white US-born people (Kindratt et al., 2024). A further risk factor for dementia that is relevant in relation to migration is social isolation. A study with Korean immigrants in America found that social isolation is associated with cognitive impairment, with loneliness as a mediator of this association (Jang et al., 2021). Furthermore, traumata and post-traumatic stress disorder (PTSD) were surveyed. Saadi et al. found both to be associated with decreased cognitive functioning in people with an Asian migration background, but not in people with a Hispanic background (Saadi et al., 2021). This is supported by a qualitative study reporting that participants with Arab, African, and Afghan migration backgrounds are convinced that stress, traumatic experiences, and mental ill-health related to their migration history are associated with dementia. Furthermore, war and experienced violence are also mentioned as affecting cognitive decline (Kaki et al., 2025).

Lifestyle factors related to migration background and dementia risk have only been studied to a limited extent. Lower odds for both smoking and alcohol consumption were found for people with a Middle Eastern or North African migration background (Kovaleva et al., 2021), whereby only lower odds of reporting alcohol consumption were found for South Asian migrants in Australia (Hamrah et al., 2025b). This was largely confirmed by the included studies in the Review of Hamrah et al. for Australia, who found obesity and alcohol consumption were a little bit lower, with smoking nearly the same or unclear across the studies, but physical inactivity was significantly higher (Hamrah et al., 2023). Overall, however, it must be noted that the factors differ greatly, much more than the regional origin (Hamrah et al., 2025a).

Migration status was also investigated, with results indicating that it is particularly relevant in relation to lifetime trajectories of SES. Among others, the number of generations since immigration is associated with the effects of cumulative deprivation on cognitive function (Haan et al., 2011). Furthermore, Moon et al. report results of immigration status as a significant moderator for the relationship between ethnicity and dementia (Moon et al., 2019).

Social aspects are an important aspect of migration and have been found in several studies. Low social cohesion was found to be a dementia risk factor by Wong et al. regarding knowing people, but more in relation to aspects of help and trust (Wong and Soong, 2024). Social isolation and loneliness were further reported as significant risk factors regarding cognitive health, whereby loneliness was found to be a potential mediator between social isolation and subjective impairment (Jang et al., 2021). Kim et al. also found a link between social isolation and cognitive health in studies of older Korean Americans, interestingly, with dietary risk as a mediator. The underlying factors named are eating alone, fewer than two meals, cooking or consumption, or not having enough money to buy food, physical difficulties obtaining food, or mouth problems (Kim et al., 2024). The importance of social participation as a key factor in dementia risk has been mentioned in other studies (Holwerda and Hoogendijk, 2020; Hamrah et al., 2023), not least confirmed by a machine learning regression with data from the Canadian Longitudinal Study, supporting the evidence of social support as a protective factor for cognitive functioning among older Chinese immigrants (Singh et al., 2023).

At the interpersonal level, there are two studies that examine the topic of dementia risk in relation to migration regarding non-migrants, the parents (Holwerda and Hoogendijk, 2020). In a first study with data from the Mexican Health and Aging study, having one or more adult children who emigrated to the US was associated with lower SES, higher depression, and greater social engagement, but no differences in odds for developing CI (Downer et al., 2018b). In a follow-up study, Torres et al. found this effect for women, but not for men, in total highlighting the migration status of family members as a highly relevant societal determinant of cognitive aging, especially in low- and middle-income countries (LMICs) (Torres et al., 2020).

At a community level, the experience of discrimination and neighborhood factors have been considered. Looking at life-course immigration, reasons for migration, acculturation, and perceived discrimination. Low levels of acculturation and perceived discrimination in particular were found to be potential risk factors for cognitive decline (Tang et al., 2023). One aspect examined several times was the neighborhood and its influence on cognition. Results suggest that while neighborhood physical disorder was significantly higher among foreign-born individuals, together with low social cohesion, neither neighborhood physical disorder nor social cohesion moderated the association between migration status and increased dementia risk (Wong and Soong, 2024). In fact, another study found that neighborhood SES was associated with poorer cognitive aging among people with migration or minority backgrounds (Weden et al., 2017). While the two studies refer to the US, a study from Denmark found that neighborhood disadvantage among refugees was not associated with dementia risk, but was associated with lower mortality (Kim et al., 2023). Finally, Goo et al. examined another aspect—living in an ethnic enclave (i.e., Chinatown). The study found that living in an ethnic enclave was a risk factor for poorer cognition, with education serving as a moderator of this effect (Guo et al., 2022).

Two studies addressed the so-called Hispanic paradox in relation to dementia and cognition. The phenomenon states that despite poorer socioeconomic factors—particularly, in this case, risk factors for dementia, such as lower education and socioeconomic disadvantage—the outcomes are not poorer. Keizos et al. find increased risk factors, but no difference in dementia risk with partwise slower decline (Kezios et al., 2023). A second study on the paradox found living in an immigrant enclave to be protective against prevalent CI among foreign-born Mexican Americans, while individual and neighborhood SES largely explained the disparities between minorities compared to US-born non-Hispanic whites (Weden et al., 2017).

Two studies examine important aspects of faith and attitudes. Faith and spirituality are particularly important for illness perception and coping strategies. Spiritual fatalism can act as a barrier, as it may hinder the understanding of illness and thus make care-seeking and preventive behavior more difficult to understand (Bridi et al., 2023a). In a subsequent study, Kaki et al. examined attitudes toward dementia among people with a migrant background. According to the qualitative study, faith and spirituality, but also trauma and anxiety, loss of self-efficacy and community support, isolation, and language barriers are relevant. At an institutional and policy level, lack of necessities, engagement with mental healthcare, and immigration policy, such as family separation and travel bans, were identified as risk factors (Kaki et al., 2025).

### 3.3 Prevention

The second research question addresses the prevention of dementia in relation to a migration background. The content of included studies can be broken down into three major topics: attitudes and beliefs, preventive behavior, and diagnosis.

Five studies examined the role of attitudes, faith, and knowledge regarding dementia and cognitive decline (Bridi et al., 2023a,b; Daniel et al., 2024; Neter and Chachashvili-Bolotin, 2022; Tran et al., 2025). The two studies by Bridi et al. analyzed attitudes and faith using two approaches at the socioecological levels and using the Leventhal Self-Regulation Model. Attitudes toward dementia on socioecological levels included the individual level, whereas individuals were convinced that mental aspects, such as stress, traumatic history from refugee experience, but also low SES and poor health, are associated with dementia risk. Also of relevance is the community level, on which shared language, religion, and culture are of importance. In this very study, those factors were mentioned as protective, since the host country was a neighboring country. Conversely, it can be assumed that those factors may be challenging and hindering preventive behavior for host countries with different backgrounds (Bridi et al., 2023b). At the community level, two other studies can be placed: Neter et al. found differences in the attitude toward Alzheimer's disease between former Soviet Union immigrants and Palestinian Citizens in Israel based on deep-rooted differences in cultural background, mainly regarding the perception of cognitive health, but also regarding the aspect of mental illness and older people. Furthermore, they report differences between the groups in preventive behavior, attributing them to socioeconomic status and human resources. The second study by Bridi et al. examined faith and its role in both illness representation and coping procedures. An important aspect of a resume is that faith is both a hindrance and a benefit, depending on its role. It can be used wisely to build coping to prevent cognitive impairment. A study interviewing 216 Latino migrants in New York about knowledge and beliefs found an overall knowledge gap, highlighting the potential for educational intervention, including cultural competency in healthcare (Tran et al., 2025). Finally, regarding attitudes and knowledge, the language scores for the host language and education were associated with knowledge of Alzheimer's disease. This was significantly improved by a single educational intervention with a faith-based health educator (Daniel et al., 2024). Another intervention was based on community engagement and dissemination with Korean American migrants. Here, effects were achieved in terms of knowledge, attitudes, and behavior. One difference here was whether people participated in person or only online, with the culturally tailored program ultimately being highlighted (van Ta Park et al., 2025).

Taken together, these studies suggest differences in attitudes and faith regarding cognitive health and preventive behavior across different cultural backgrounds. However, while it partially hinders examination and perception of cognitive health, spiritual practices can be used to build resilience and target prevention strategies related to this background.

Regarding preventive behavior, four of the included studies contribute: Neter et al. found a strong correlation between Internet use and knowledge toward Alzheimer's disease and preventive behavior. Building knowledge about AD, including risk factors, needs to take into account cultural attitudes, but by doing so, the promotion of dementia risk reduction is possible (Neter and Chachashvili-Bolotin, 2022). This was done in a study with Afro-Caribbeans living in a rural area in the USA. The pre-post study showed a significant increase in knowledge about AD and prevention after a single intervention, which consisted of a review and discussion of the participants' basic knowledge about AD with a faith-based health educator, including health-promoting factors such as social engagement, exercises, managing diabetes, and hypertension (Daniel et al., 2024). Two study protocols with similar ideas were found. The first protocol by Teixeira-Santos et al. describes a planned two-armed intervention with one a health-promoting program and a mindfulness-based stress reduction program over 8 weeks with migrant individuals (Teixeira-Santos et al., 2023). A second protocol describes the MIND-PRO (The Mobile Health (mHealth) Intervention for Dementia Prevention through lifestyle Optimization), a self-managing lifestyle modification intervention including remote coaching for participants with lower socio-economic status and/or migration background (van der Endt et al., 2025). No results were found yet. Completing a review of Adult Day Service Centers (ADS) is to name, which mentions cognitive health and dementia only in passing. Nevertheless, ADS were effective in supporting individual's health most effectively by culturally based components. The use and efficiency differed by functional impairment, gender, and degree of loneliness. ADS are therefore relevant for primary prevention of dementia, as they address the psychosocial needs of individuals. ADS enable social participation, integrating individuals into social structures, and ensure healthy nutrition and physical activity. Important for participation were transportation, bilingual nurses, peer support, and cultural activities (Sadarangani and Murali, 2018).

What stands out is a theoretical review about the design and implementation of culturally safe dementia risk reduction strategies for immigrant and ethno-culturally diverse women. None of the included 17 studies focused on women only, a group at enhanced risk due to multiple interacting factors, compared to men. However, based on the evidence, the authors recommend focusing on in-person meetings, including didactic lectures and interactive discussions, using videos and follow-up materials, and participants' first language. Furthermore, involving representatives from a community and using community organizations can be beneficial to improve dementia knowledge (Iziduh et al., 2025).

Additionally, one study examined diagnosis from the perspective of General Practitioners, which is crucial for secondary prevention. Almost all participants who answered the questionnaire reported barriers at least once in diagnosing dementia for individuals with a migrant background (96%). Most common reasons were uncertainties, language barriers, and information deficits in patients, as well as a lack of acceptance and shameful interactions (Tillmann et al., 2019).

### 3.4 Most marginalized individuals

There was hardly any evidence about risk, risk factors, or prevention of dementia for more marginalized individuals with a migrant background, such as asylum seekers, undocumented and irregular migrants, and refugees. Two studies were initially included (Simon et al., 2023; Hamrah et al., 2023). Both studies are reviews, and neither reports results for the group of interest. In the review by Hamrah et al., the terms refugees, asylum seekers, and migrants were used interchangeably without further stratification. Only two of the included studies of the review report results regarding asylum seekers, indicating a higher prevalence of depression (Lies et al., 2019; Silove et al., 2007). The perspective review by Simon et al. raises ambiguity about the inclusion of undocumented individuals in the term *migrants*, making it harder to analyze the diverse life situations of individuals with a migrant background. One study certainly focuses on undocumented migrants in the U.S., but after returning to Mexico as their country of origin. Undocumented migrants were classified as not being reported as permanent residents or citizens, and not being reported as contributing to the U.S. Social Security system or having a Social Security number. This was found to be a risk factor due to migration experience, especially when working in the informal sector after return (Oaxaca Carrasco et al., 2025). According to a situation, implementation is seen differently as well. The authors stressed that there is an urgent need to include and focus on undocumented people, in this case, undocumented Brazilians living in the US (Simon et al., 2023).

## 4 Discussion

Following the main research question about risk and potential prevention of dementia in relation to migrant background, we were able to amalgamate previous research, subdivided into three topics, whereby mainly evidence about risk, risk factors, and prevention contributes so far. Discussing the findings, however, it is necessary to acknowledge a substantial heterogeneity across migrant populations and the methodological inconsistencies in the current evidence, although a significant amount, so far, is coming from the U.S., with a focus on individuals of Hispanic background. This must be kept in mind when interpreting the results and will be discussed in more depth below.

At an individual level, there are known risk factors, but they are more pronounced among individuals with a migration background. In summary, these include older age (at migration), gender, but above all, socio-economic factors and language barriers.

Higher age at migration has been repeatedly associated with increased risk of cognitive decline (Luo et al., 2023; Vásquez et al., 2022). This result is in line with other studies about age at migration and health, for example, regarding self-reported health (Gubernskaya, 2015). This may have various reasons to consider when thinking about prevention, ranging from differences in risk profiles, health literacy, and healthcare before migration to more limited access to social participation, healthcare, and diagnoses after migration (Gubernskaya, 2015).

Language and language proficiency were also found to increase risk for cognitive decline in studies regarding migrant backgrounds. This is associated with an increasing tendency toward depressive symptoms, more difficult social participation or acculturation, but also a more difficult diagnosis of cognitive decline (Daniel et al., 2024; Kovaleva et al., 2021; Franco and Choi, 2020). All of those studies were considered in the U.S., where the samples were quite heterogeneous and differed in their country of origin. This substantiates the universality of language as a barrier to social inclusion, access to the health care system, and overall health-promoting factors for people with a migrant background. Language is a risk factor that clearly distinguishes individuals with a migrant background from the host population. Beyond dementia risk, language is seen as a serious barrier to healthy aging for people with a migrant background (Pot et al., 2020).

Our research found several studies on already known risk factors, according to the Lancet Commission's current list of fourteen factors (Livingston et al., 2024). Education, depression, diabetes, hypertension, and social isolation seem to be of special relevance in relation to migration background based on our results. Education must be given a particularly important role within the association between migration or a migrant background and dementia. Thus, separate from the known effect on dementia risk, we found education mediating and moderating the impact of other migration-related risk factors. Higher education was found to lower the positive association between age at migration and cognitive impairment (Downer et al., 2018a) but also explained the disadvantage of living in so-called ethnic enclaves (Guo et al., 2022). While both results indicate that social participation and acculturation as protective factors for mental and cognitive health, they further highlight the assumption that lower education even strengthens the already important aspects of aging and health among individuals with a migrant background. Those include a more disadvantaged SES, language proficiency, or lower health literacy, but also health-seeking behavior (Kristiansen et al., 2016). Regarding physical health, diabetes and hypertension were found to be more prevalent in individuals with a migrant background, together with a higher risk for obesity and hearing loss (Kindratt et al., 2024; Hamrah et al., 2023). The link between physical health and migration has already been well-researched, so we will not go into it further here. However, to point to elaborated strategies, we refer here to a publication by Matlin et al., who propose an agenda of solutions and emphasize the “*right to health of all individuals and the centrality of this principle in the design and implementation of policies and programs at global, regional, national and local levels*” (Matlin et al., 2018).

In addition to physical health, the mental health aspects of people with a migrant background were often mentioned. Depending on migration status, migration experience, and other migration-related factors, there is an increase in depression, anxiety disorders, trauma, and PTSD. All of these factors can be seen as risk factors of dementia. A special attention to this is therefore not only relevant for the respective diseases, but also for the importance of the risk of dementia due to the impact and possible comorbidity. From an intersectional perspective, as with other risk factors, migration can be seen as a reinforcing factor or a moderator.

Regarding the second research objective about prevention and its potential in relation to migration background, a few studies examined the role of attitudes and faith. These results are significant in at least two major respects. First, spirituality might serve as a barrier to implementing certain health-providing behaviors, i.e., due to spiritual fatalism. On the other hand, faith and spirituality are found to be resources for preventive behavior and coping procedures, for example, through trust and gratitude. However, both proclivities are of big relevance for prevention when considering modern approaches like the ‘Behavioral and Cultural Insights' (BCI), which has been recommended by the WHO (World Health Organization, 2023). BCI can be used to plan and implement preventive strategies based on specific health-related behaviors regarding psychological, social, or cultural factors. One aspect of BCI is that local contexts and needs of specific target groups are of relevance (Loss et al., 2025). Although there is first evidence about the effects of different spiritualities and belief systems, concentrated evidence must be expanded to be included in the development of interventions that are behavior-based and based on social circumstances.

Two studies that emerged from this review describe promising approaches to the prevention of dementia specified to people with a migrant background, referring to risk factors from research question one. This is primarily Internet use, which was found to increase knowledge about dementia (Neter and Chachashvili-Bolotin, 2022). A part of the possibility to increase knowledge of dementia, which is an important step toward risk reduction (van Asbroeck et al., 2021), a promising approach might be applicable in specific cases. One example for the successful use of an application for mental health among individuals with a migrant background is Sanadak, a self-help app for migrants from Syria to cope with posttraumatic stress (Röhr et al., 2021). There are already apps for dementia knowledge and prevention (Chelberg et al., 2022) and more are planned (van Asbroeck et al., 2023). Including aspects such as different languages or taking possible cultural and religious aspects into account, this could be a food-first, low-threshold approach to communicate knowledge about dementia and prevention.

A second approach that could facilitate initial knowledge and prevention options, as well as possible access, is the mentioned adult day service centers (ADS). ADS may offer low-threshold, community-based support that can promote cognitive and social stimulation, which are key components for dementia prevention. For older adults with a migrant background, culturally sensitive ADS may help reduce social isolation or improve access to preventive information, as discussed by Sadarangani and Murali (2018).

Only three studies were included regarding research question three, addressing dementia risk and prevention in general among highly marginalized migrant populations, such as asylum seekers, undocumented individuals, or people with irregular migration status. Importantly, except for one study, which, however, refers to returned migrants, neither study presented empirical data on dementia-related outcomes. Instead, there is an urgent need for research in this area. This striking gap shows the practical challenge of reaching these groups, as well as broader patterns of structural invisibility in health research and policy. This is, among others, also found in general health research (Young and Madrigal, 2017) or research regarding health care (Woodward et al., 2014; Hacker et al., 2015).

A specific aim of this scoping review was to summarize first previous evidence to detect possible research gaps. As mentioned above, it is important to bear in mind the general heterogeneity within and between migrant populations. Furthermore, a significant portion of the included studies stems from the United States, with a strong focus on individuals of Hispanic background. These studies offer important insights; however, they reflect a specific socio-political and healthcare context of the U.S. and thus may not be easily transferable to a European or other global setting. This regional concentration of the research so far highlights a lack of research on other migrant populations, including those of Middle Eastern, African, or Eastern European origin, other host countries, and particularly individuals with precarious or undocumented status.

Furthermore, even if we included a working definition and checked the included studies, respectively, there is considerable variation in how migrant background is defined and operationalized—for instance, by country of birth, ethnicity, or self-identification. Furthermore, there are formative differences in the circumstances and reasons for migration. This is highly relevant, especially about the factors explained above, as reasons for migration or origin may have a major influence on individual risk factors, resources, or social participation. Some of the findings might certainly be transferable, but the contexts differ in terms of countries of origin, experiences of migration in terms of circumstances, contexts, and experiences in host countries. It is therefore essential that research is continued and intensified, adapted to relevant circumstances. It is relevant to consider several factors when investigating this topic, including differences of a pre-, peri-, or post-migrant nature, or migration as a possible moderator of the known risk factors and their influence on dementia risk. Hossin (2020) discussed different theoretical frameworks with the conclusion that looking only at acculturation factors is not enough, just like the social determinants of health framework in relation to migration. Due to increasing migration and a big heterogeneity of reasons for migration, migration status, and differences among individuals with migrant backgrounds, an intersectional approach is needed to do justice to as many relevant factors and levels as possible.

### 4.1 Limitations and strengths

Conducting this scoping review, we provide a double-screened synthesis of peer-reviewed literature on the risk and prevention of dementia in migrant populations, in the hopes of supporting future research targeting risk factors that may be higher in migrant populations and of implementing targeted and effective prevention. To our knowledge, this is the first scoping review to combine risk and prevention for dementia in relation to migration background. In doing so, however, there are some limitations, some of which are content-related. First, we did not assess the methodological quality of the risk-of-bias assessment of the included studies. However, this is typical for a scoping review and is based on the wide range of study types (Peters et al., 2022). Second, following this argument, there was significant heterogeneity of study designs, populations, and definitions of “migrant background,” which limits the comparability of findings, as already discussed. Third, our search was restricted to English- and German-language publications, excluding relevant studies in other languages, which is particularly relevant given the topic. Furthermore, a majority of the included studies were conducted in the US, with a strong focus on individuals of Hispanic background. This limits the universality of findings to other migrant populations, particularly in Europe. Finally, some studies highlighted challenges in ensuring the cultural and linguistic appropriateness of research instruments or procedures. These challenges may affect the validity of both risk and outcome measurements in migrant populations or might lead to selection biases.

## 5 Conclusion

With a growing population of aging Individuals with migrant backgrounds, addressing health inequities has to become a public health priority, also in dementia risk and prevention. In this sense, and supported by our results, migration should not merely be seen as a background variable, but as a dynamic modifier of dementia risk (factors). This scoping review highlights relevant factors and first approaches to targeted prevention, but key gaps in the literature: while several studies have identified relevant risk factors and possible prevention strategies, few address how these interact. Focusing on migrant background, evidence suggests that tailored dementia prevention must operate not only at an individual level but also at a community and societal level, by reducing barriers, be it of language matter, lack of cultural understanding and offers, to healthcare, but above all discrimination. A central conclusion is that an analysis of the heterogeneity of populations and definitions must come first. This is essential for further research, whereby the difficulty of generalizability must be addressed directly. However, future prevention research should prioritize its implementation with attention to migration status and local contexts. Strengthening prevention efforts through inclusive public health strategies will be essential to reducing inequalities in dementia risk and ensuring equitable access to care and healthy aging for all.

## Data Availability

The original contributions presented in the study are included in the article/Supplementary material, further inquiries can be directed to the corresponding author.
